# T-cell infiltration in the central nervous system and their association with brain calcification in *Slc20a2*-deficient mice

**DOI:** 10.3389/fnmol.2023.1073723

**Published:** 2023-01-20

**Authors:** Yi Zhang, Yaqiong Ren, Yueni Zhang, Ying Li, Chao Xu, Ziyue Peng, Ying Jia, Shupei Qiao, Zitong Zhang, Lei Shi

**Affiliations:** ^1^Human Molecular Genetics Group, NHC Key Laboratory of Molecular Probes and Targeted Diagnosis and Therapy, The Fourth Affiliated Hospital of Harbin Medical University, Harbin, China; ^2^Department of Medical Genetics, College of Basic Medical Sciences, Harbin Medical University, Harbin, China; ^3^Department of Child and Adolescent Health, School of Public Health, Harbin Medical University, Harbin, China; ^4^Department of Pediatrics, The Second Affiliated Hospital of Harbin Medical University, Harbin, China

**Keywords:** brain calcification, *Slc20a2*, T-cell, blood–brain barrier, permeability, transcytosis, FTY720

## Abstract

Primary familial brain calcification (PFBC) is a rare neurodegenerative and neuropsychiatric disorder characterized by bilateral symmetric intracranial calcification along the microvessels or inside neuronal cells in the basal ganglia, thalamus, and cerebellum. *Slc20a2* homozygous (HO) knockout mice are the most commonly used model to simulate the brain calcification phenotype observed in human patients. However, the cellular and molecular mechanisms related to brain calcification, particularly at the early stage much prior to the emergence of brain calcification, remain largely unknown. In this study, we quantified the central nervous system (CNS)-infiltrating T-cells of different age groups of *Slc20a2*-HO and matched wild type mice and found CD45^+^CD3^+^ T-cells to be significantly increased in the brain parenchyma, even in the pre-calcification stage of 1-month-old -HO mice. The accumulation of the CD3^+^ T-cells appeared to be associated with the severity of brain calcification. Further immunophenotyping revealed that the two main subtypes that had increased in the brain were CD3^+^ CD4^−^ CD8^–^ and CD3^+^ CD4^+^ T-cells. The expression of endothelial cell (EC) adhesion molecules increased, while that of tight and adherents junction proteins decreased, providing the molecular precondition for T-cell recruitment to ECs and paracellular migration into the brain. The fusion of lymphocytes and EC membranes and transcellular migration of CD3-related gold particles were captured, suggesting enhancement of transcytosis in the brain ECs. Exogenous fluorescent tracers and endogenous IgG and albumin leakage also revealed an impairment of transcellular pathway in the ECs. FTY720 significantly alleviated brain calcification, probably by reducing T-cell infiltration, modulating neuroinflammation and ossification process, and enhancing the autophagy and phagocytosis of CNS-resident immune cells. This study clearly demonstrated CNS-infiltrating T-cells to be associated with the progression of brain calcification. Impairment of blood–brain barrier (BBB) permeability, which was closely related to T-cell invasion into the CNS, could be explained by the BBB alterations of an increase in the paracellular and transcellular pathways of brain ECs. FTY720 was found to be a potential drug to protect patients from PFBC-related lesions in the future.

## Introduction

Primary familial brain calcification (PFBC; OMIM#213600), also known as Fahr’s disease, is a rare neurodegenerative disorder characterized by bilateral and symmetric calcification along the microvessels or inside neuronal cells in the basal ganglia, thalamus, and cerebellum. This disease is accompanied by multiple neurological manifestations such as movement disorders, cognitive impairment, and psychiatric signs, which commence after the age of 40 years (Tadic et al., 2015; Grangeon et al., 2019; Xu et al., 2022). Primary familial brain calcification is caused by the loss-of-function (LOF) variants, either in a dominant or recessive inheritance pattern, in one of seven genes in humans: solute carrier family 20 member 2 (*SLC20A2*; Wang et al., 2012), platelet derived growth factor receptor beta (*PDGFRB*; Nicolas et al., 2013), platelet derived growth factor subunit B (*PDGFB*; Keller et al., 2013), xenotropic and polytropic retrovirus receptor 1 (*XPR1*; Legati et al., 2015), myogenesis regulating glycosidase (putative) (*MYORG*; Yao et al., 2018), junctional adhesion molecule 2 (*JAM2*; Cen et al., 2020; Schottlaender et al., 2020) and cytidine/uridine monophosphate kinase 2 (*CMPK2*; Zhao et al., 2022).

*Slc20a2* encodes a multi-transmembrane type III sodium-dependent phosphate (Pi) cotransporter 2 (Pit-2), which is essential for regulating Pi homeostasis in the cerebrospinal fluid (CSF) and brain parenchyma. Its functional deficiency impedes inward Pi transport into brain endothelial cells (ECs) or smooth muscle cells (SMCs), leading to paracellular Pi accumulation (Wallingford et al., 2017). CSF-Pi levels are significantly elevated in patients with *SLC20A2*-related PFBC (Paucar et al., 2017; Hozumi et al., 2018) and in *Slc20a2* homozygous (HO) knockout mice (Jensen et al., 2016; Wallingford et al., 2017). Excess extracellular Pi is known to cause tissue toxicity (Razzaque, 2011; Hong et al., 2015) and is associated with neuroinflammation (Brown, 2020), so as the accumulation of intracellular Pi (Legati et al., 2015; Zhao et al., 2022). The central nervous system (CNS)-resident immune cells, particularly microglia, macrophages, and astrocytes, are important sources of neuroinflammatory cytokines. Among these, CD45^+^ microglia (Keller et al., 2013), PDPN^+^, LCN2^+^, or C3^+^ neurotoxic reactive astrocytes (Zarb et al., 2019), along with novel calcification-associated microglia (Zarb et al., 2021), are known to significantly increase and cluster around the perivascular spaces of calcified microvessels in PFBC mice.

The blood–brain barrier (BBB) serves as a highly selective, restrictive, and dynamic monolayer barrier for peripheral immune cells and foreign immunogens while also overseeing the CNS immune surveillance and brain homeostasis and maintaining the normal physiological roles of the CNS (Zlokovic, 2008; Abbott et al., 2010). Microvascular ECs, the core components of the BBB, express cell adhesion molecules (CAMs) to restrict peripheral immune cell crawling and adherence and possess specific cell junction proteins to prevent harmful immunogen extravasation and maintain beneficial exchange through paracellular and transcellular pathways under physiological conditions (Wilson et al., 2010; Engelhardt and Ransohoff, 2012; Pulgar, 2018). Maintaining a low rate of endocytosis and transcytosis in the BBB also contributes to normal CNS function. Transcellular pathway could be mediated by clathrin or caveolin-1 (Cav-1) expressed on ECs (Andreone et al., 2017; Ayloo and Gu, 2019). The proto-oncogene tyrosine-protein kinases, Src and Cav-1, participate in the transcytosis process, where Src regulates the formation of endocytic vesicles by phosphorylating Cav-1, the primary structural component of caveolae (Parton et al., 1994; Kim et al., 2009).

Peripheral immune cell infiltration in the CNS has been reported as coinciding with neurological disorders, such as Alzheimer’s disease, multiple sclerosis, and stroke (Ellwardt et al., 2016; Liebner et al., 2018), among which CD4^+^ and CD8^+^ T-cells account for the main subpopulations. FTY720 (Fingolimod) is a sphingosine-1-phosphate receptor modulator, which inhibits egress of lymphocytes from lymph nodes, potentially reducing trafficking of T-cells into the CNS (Mandala et al., 2002; Cohen and Chun, 2011), and was utilized to inhibit the circulation of peripheral T-cells to treat CNS autoimmune diseases such as multiple sclerosis (Chun et al., 2019). In *Pdgfb*^ret/ret^ PFBC mice, CD45^hi^ leukocyte infiltration modified the course of neuroinflammation, and the infiltration and neuroinflammation could be alleviated by FTY720 (Torok et al., 2021). However, in *Slc20a2*-PFBC mice, the cellular and molecular mechanisms related to brain calcification, particularly in a high-Pi microenvironment or areas of microcalcification, remain largely unknown.

## Materials and methods

### Mouse model

*Slc20a2*^tm1a(EUCOMM)Wtsi^ mice were purchased from the European Mouse Mutant Archive (Munich, Germany; Skarnes et al., 2011). The mice were maintained under specific pathogen-free conditions and allowed free access to water and chow under a light–dark cycle of 12 h. Heterozygous *Slc20a2* mice were cross-mated to generate wild-type (WT) and HO mice. Littermates were used in subsequent experiments. All research procedures related to mouse models were scrutinized and approved by the Ethics Committee of the Fourth Affiliated Hospital of Harbin Medical University.

### Immunofluorescence

Brain tissues embedded in optimal cutting temperature media were cut into 5 μm sections and stored at –80°C. Brain sections were fixed with 4% paraformaldehyde (PFA) for 10 min on ice and blocked with blocking buffer [5% normal goat/donkey serum, 1% bovine serum albumin (BSA), 0.3% Triton X-100, and 0.3% glycine in 0.01 M phosphate-buffered saline (PBS)] for at least 1 h at 25°C. The sections were incubated with primary antibodies overnight at 4°C, followed by incubation with secondary antibodies conjugated to Alexa Fluor 488 or 594. Images were obtained using a confocal microscope (C2 confocal system, Nikon, Japan). All the antibodies and immunofluorescence parameters are listed in Supplementary Table S1.

### Leukocyte isolation and flow cytometry

The mice were anesthetized and transcardially perfused with ice-cold Hank’s balanced salt solution (Ca/Mg-free). The brain was immediately isolated and homogenized mechanically through a 100 μm cell strainer, followed by digestion with Liberase (Roche, 5401020001) for 1 h at 37°C. The cell suspension was passed through a 70 μm cell strainer and rinsed with Hank’s balanced salt solution (Ca/Mg-free) containing 10% fetal bovine serum with or without DNase I (Roche, 10104159001). After density gradient centrifugation using 25% Percoll to remove myelin and cell debris, the cell pellet was resuspended in Hank’s balanced salt solution (Ca/Mg-free) containing 10% fetal bovine serum. A single-cell suspension was prepared for flow cytometry with a BD Accuri C6 Plus instrument. All antibodies and additional information for flow cytometric analysis are listed in Supplementary Table S1.

### Micro-computed tomography

Micro-computed tomography (IRIS PET/CT imaging system, Inviscan, France) was applied to show the distribution of calcifications in the brains of the mice. Mice were deeply anesthetized with isoflurane for approximately 5 min before the experiment. They were then placed on the imaging stage under anesthesia with isoflurane gas during the entire imaging process. A total of 1,945 coronal plane images were obtained from each mouse. Approximately 70 images showed calcification in the hypothalamus and midbrain of the brains of 12-month-old *Slc20a2*-HO mice.

### Brain microvessel isolation

Mice were anesthetized with an intraperitoneal injection of 20% Ulatan (70 μl/10 g body weight). The entire brain was isolated from the skull and washed in cold isotonic sucrose buffer (0.32 mol/l sucrose, 3 mmol/l HEPES in distilled water, pH = 7.40). Each brain sample was homogenized in 5 ml sucrose buffer using a Dounce homogenizer, followed by centrifugation at 4500 × *g* for 10 min at 4°C. After removing the supernatant, the precipitate was resuspended in sucrose buffer and centrifuged again. The final pellet was resuspended in 2 ml sucrose buffer and passed through a 40 μm nylon mesh. Brain microvessels were retained on the mesh and eluted with ice-cold 0.01 M PBS (pH = 7.40) for collection. Tissue was obtained by centrifugation, and RIPA (YEASEN, 20115ES60), cell lysis buffer (CST, 9803), or TRIzol™ Reagent (Invitrogen, 15596018) were added to extract protein or RNA.

### Quantitative real-time polymerase chain reaction (qRT-PCR)

Total RNA was extracted from brain microvessels using TRIzol^™^ Reagent (Invitrogen, 15596018) and then reverse-transcribed into first-strand complementary DNA using the SuperScript^™^ IV RT-PCR System (Invitrogen, 12594025). qRT-PCR was performed utilizing LightCycler^®^ 480 SYBR Green I Master Mix (Roche, 04887352001) on a Roche 480 instrument. All assays were performed in triplicate, and the levels of each gene were normalized to those of the housekeeping gene *Hprt*. All primer sequences and additional information for qRT-PCR are listed in Supplementary Table S2.

### Western blotting

Total protein was extracted from brain microvessels and quantified using a BCA Protein Assay Kit (Solarbio, PC0020). After adjusting to the consistent concentration, proteins were separated by sodium dodecyl sulfate–polyacrylamide gel electrophoresis (Bio-Rad, 161-0183), followed by the transfer of proteins from the gels to polyvinylidene difluoride membranes. 5% non-fat milk was used to avoid non-specific protein binding for 1 h at 25°C, followed by overnight incubation with primary antibodies diluted in 5% non-fat milk or 5% BSA at 4°C. After incubation with secondary antibodies, an enhanced chemiluminescence substrate was added to the membranes to highlight the target bands. All the antibodies used and additional information for western blotting are listed in Supplementary Table S1.

### Blood–brain barrier (BBB) permeability/leakage detection

Mice received an intraperitoneal injection of 2% Evans Blue (Sigma-Aldrich, E2129) in normal saline (4 ml/kg body weight) 2 h before sacrifice or a tail vein injection of 100 μl of 3 or 70 kDa tetramethylrhodamine-dextran (2 and 10 mg/ml, Invitrogen, D7162 and D1818, respectively) 1 h before sacrifice. Subsequently, the mice were transcardially perfused with 30 ml of PBS, followed by 60 ml of ice-cold 4% PFA. Brain tissue was post-fixed in 4% PFA for 4 h, dehydrated in 30% sucrose overnight at 4°C, and embedded in an optimal cutting temperature medium on dry ice. Brain sections were cut into 10- or 30-μm-thick sections for Evans Blue or tetramethylrhodamine-dextran staining and visualized using a Nikon C2 confocal microscope at 555- or 580-nm emission, respectively.

### Immunoelectron microscopy

Mice were transcardially perfused with a mixture of 0.2% glutaraldehyde and 4% PFA in 0.1 M phosphate buffer under deep anesthesia. The brain’s basal ganglia, thalamus, and hypothalamus regions were quickly removed and cut into 1-mm^3^ pieces, placed into 0.2% glutaraldehyde in 0.1 M phosphate buffer, and fixed for 2 h at 25°C. After dehydration, embedding, and sectioning, the sections were incubated with 1% BSA blocking buffer for 30 min at 25°C and then incubated with rabbit anti-CD3 antibody (1:50; Abcam, ab5690) in 1% BSA overnight at 4°C. After washing, the sections were incubated with 10 nm gold-labeled goat anti-rabbit IgG (1:50; Sigma-Aldrich, G7402) and stained with 1% aqueous uranyl acetate for 20 min, followed by lead citrate for 30 s. The sections were photographed under a HITACHI HT7800 electron microscope.

### FTY720 administration

*Slc20a2*-HO mice, aged 1.5 months, were given 0.5 mg/kg FTY720 (Selleck, S5002) diluted in dimethyl sulfoxide or dimethyl sulfoxide alone *via* intraperitoneal administration three times a week, once every 2 days. After 12 weeks, the mice were transcardially perfused with 30 ml of PBS, and half of the brain was extracted for RNA using TRIzol^™^ Reagent (Invitrogen, 15596018), while the other half was fixed in ice-cold 4% PFA for 16 h. After dehydration in 30% sucrose at 4°C overnight, the brains were collected for further experiments.

### von Kossa staining

von Kossa staining was applied to detect phosphate ions (PO_4_^3−^). Following the staining protocol (Abcam, ab150687), the sections were dewaxed, rehydrated, and incubated with 5% silver nitrate for 45 min under ultraviolet rays. After rinsing several times with fresh distilled water, the sections were incubated with 5% sodium thiosulfate solution for 10 min to wash the unreacted silver ions, followed by a nuclear fast red solution.

### RNA sequencing bioinformatic analysis

Brain RNA samples from mice administered the placebo/DMSO (*n* = 4), and FTY720 (*n* = 6) were collected for RNA sequencing. RNA libraries for RNA-seq were prepared using NEBNext^®^ Ultra^™^ RNA Library Prep Kit for Illumina^®^ following manufacturer’s protocol. Illumina Casava 1.8 software was applied for basecalling. Raw data of fastq format were firstly processed through in-house Perl scripts. Reference genome GRCm38/mm10 and gene model annotation files were downloaded and then built and paired-end clean reads were aligned to the reference genome using Hisat2 v2.0.5. Differentially expressed genes (DEGs) were defined as the criterion *p* < 0.05 and protein-coding function. A volcano plot was constructed to map all DEGs using the Ggplot2 package (version 3.0.3). Gene Ontology (GO) and Kyoto Encyclopedia of Genes and Genomes (KEGG) enrichment analyses were applied to identify the DEG function, with the cut-off criteria of *p* < 0.01 and 0.05. Results were presented using the clusterProfiler package (version 3.0.3).

### Statistical analysis

All data are presented as individual mean ± SD or SEM. CD45^+^CD3^+^ cells in the brains of 1-, 3.5-, and 8-month-old mice were counted in ten equally spaced sections covering whole brain with at least three biological replicates. The composition and percentage of T-cells in the brain parenchyma of five groups of 12-month-old mice were analyzed. For the quantitative analysis of Evans Blue leakage, under the identical conditions of sample processing and capture parameters, the average fluorescence intensity was calculated on three whole brain slices of each mouse using ImageJ (version 1.53c). For the evaluation of BBB integrity related with astrocytes and pericytes, AQP4/PDGF-Rβ and CD31 double positive areas were determined using the ImageJ area measurement tool as the percentage of AQP4-or PDGF-Rβ-positive fluorescent areas covering CD31-positive areas with five equally spaced sections in the 1- and 3.5-month-old mice. For statistical analysis, three visual fields were randomly selected in the cortex, basal ganglia, and midbrain regions of each brain slice. Simultaneously, the fluorescence intensity of AQP4- and PDGF-Rβ-positive areas was statistically analyzed. Shapiro–Wilk test and Levene’s test were used to examine the normality of the data and equality of variance, respectively. All data were compared using unpaired t-test except for the data of the expression of proteins from brain microvessels using paired t-test. Statistical significance was set at *p* < 0.05. ^*^, ^**^, ^***^, ^****^, and n.s. represent *p* < 0.05, *p* < 0.01, *p* < 0.001, *p* < 0.0001, and “not statistically significant,” respectively.

## Results

### T-cell infiltration positively correlated with brain calcification and aging

**Figure 1:**
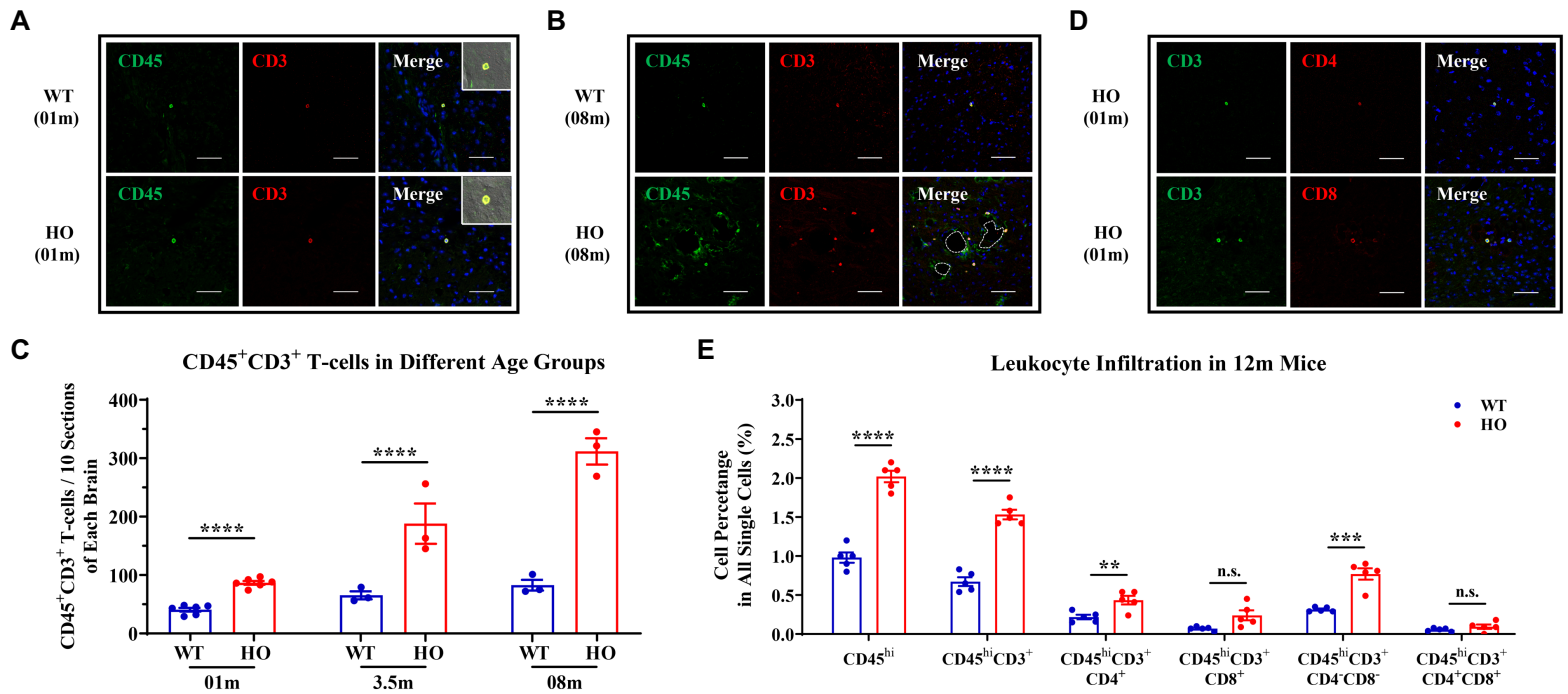
Increased CNS Infiltration of T-Cells and Their Subpopulations in *Slc20a2*-HO Mice. **(A)** CD45^+^ CD3^+^ T-cells in the brain parenchyma of 1-month-old *Slc20a2*-WT and -HO mice. **(B)** Accumulation of T-cells around the calcification in 8-month-old -HO mice. Dashed lines indicate the outline of the calcified spots. **(C)** Absolute number of T-cells in the brain of -WT and -HO mice at 1-, 3.5-, and 8-month-old (Data are shown as mean ± SEM; 1 month, *n* = 6 pairs; 3.5 months, *n* = 3 pairs; 8 months, *n* = 3 pairs; 10 equally spaced sections covering entire brain with three biological replicates). **(D)** CD4^+^ and CD8^+^ T-cells in brain parenchyma of 1-month-old -HO mice. **(E)** Leukocyte immunophenotyping in 12-month-old -WT and -HO mice. Scale bar, 40 μm. ^****^p < 0.0001; ^***^p < 0.001; ^**^p < 0.01; n.s., not statistically significant.

To determine the presence of T-cell infiltration in the brain parenchyma of *Slc20a2*-HO mice at the pre-calcification stage, CD45 and CD3 markers were used to label T-cells by immunofluorescence. CD45^+^CD3^+^ T-cells emerged in the brain parenchyma of -HO and -WT mice at the age of 1 month (Figure 1A). To explore the relationship between CNS-infiltrating T-cells and calcification severity, T-cells were quantified in 3.5- and 8-month-old mice (Figures 1B,C), representing the pathological stages of slight and moderate calcification (Wallingford et al., 2017; Jensen et al., 2018; Ren et al., 2021). There was a significant increase in the number of CD45^+^CD3^+^ T-cells in -HO mice among the three age groups, with approximately a 2-, 3-, and 4-fold increase, compared to that in -WT mice, respectively (Figure 1C). More T-cells appeared in the brains of mice with severe brain calcification and older age, suggesting that T-cells infiltrated persistently with increasing calcification and age (Figure 1C; Supplementary Figure S1A). Notably, most infiltrating T-cells clustered around the calcified regions (Figure 1B; Supplementary Figure S1A).

### Central nervous system (CNS)-infiltrating T-cells mainly consisted of CD4^+^ and CD4^−^CD8-T-cell subsets

Consistent with other neuroinflammatory diseases, both CD4^+^ and CD8^+^ cells, two classic subsets of CD3^+^ T-cells, were present in the brain parenchyma of 1-month-old *Slc20a2*-HO mice (Figure 1D), and these were hardly observed in -WT mice of the same age. To further precisely determine the subpopulation and proportion of infiltrated T-cells associated with brain calcification, flow cytometric analysis was performed on the brains of 12-month-old mouse pairs, as well as 14- and 21-month-old -HO mice. Firstly, micro-computed tomography had demonstrated the presence of calcified plaques in the hypothalamus, basal ganglia, and midbrain of 12-month-old -HO mice (Supplementary Figure S1B), which was consistent with previous reports (Wallingford et al., 2017; Jensen et al., 2018; Ren et al., 2021). Flow cytometric analysis confirmed an increase in CD45^hi^ and CD3^+^ cells in the brains of 12-month-old -HO, compared with -WT mice (proportion of all single cells = CD45^hi^: WT: 0.980%, HO: 2.020%, *p* < 0.0001; CD3^+^ cells = WT: 0.672%, HO: 1.532%, *p* < 0.0001). In the CD3^+^ T-cell subpopulation, CD4^+^ and CD4–CD8 double-negative T (DNT) cells significantly increased in the brains of -HO mice (proportion of all single cells = CD4^+^ cells: WT: 0.218%, HO: 0.434%, *p* = 0.0086; DNT cells = WT: 0.314%, HO: 0.770%, *p* = 0.0003). However, CD8^+^ and CD4–CD8 double-positive T (DPT) subsets were not significantly different between the -HO and -WT groups (proportion of all single cells = CD8^+^ cells: WT: 0.070%, HO: 0.240%, *p* = 0.0541; DPT cells = WT: 0.056%, HO: 0.094%, *p* = 0.2438; Figure 1E). Furthermore, CD3^+^CD4^+^ and DNT subpopulations were increased in the older mouse groups (14- and 21-month-old), and CD45^hi^ cells were also noted to increase with age (Figure 1E; Supplementary Figure S1C).

### T-cells most likely travel to the brain parenchyma *via* the paracellular pathway

**Figure 2:**
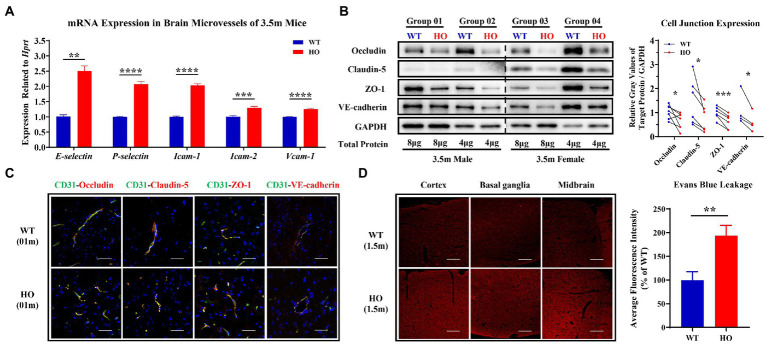
Expression and distribution pattern of core adhesion molecules and junction proteins and the blood–brain barrier (BBB) permeability of paracellular pathway. **(A)** The relatively increased expression of selectin and cell adhesion molecules (CAMs) in brain microvessels of 3.5-month-old *Slc20a2*-HO compared to that of -WT mice (*n* = 3 pairs). **(B)** Decreased protein expression of TJs (occludin, claudin-5, and ZO-1) and AJ (VE-cadherin) in brain microvessels of 3.5-month-old -HO compared to that of -WT mice. **(C)** No difference in localization, morphology, and polarization of TJs and AJ proteins in 1-month-old -WT and -HO mice. Brain microvessels visualized by CD31. **(D)** Evans blue have slightly increased entry into the brain parenchyma of -HO compared to -WT mice at the age of 1.5 months. Scale bars, 40 μm **(C)**, 80 μm **(D)**. ^****^p < 0.0001; ^***^p < 0.001; ^**^p < 0.01; ^*^p < 0.05.

To explore whether T-cells have the possibility of entry into the CNS through the paracellular pathway, we evaluated the expression of EC permeability-associated proteins including selectins, CAMs and cell junctions. In isolated brain microvessels of 3.5-month-old *Slc20a2*-HO mice, the expression levels of selectin (*E-selectin* and *P-selectin*) and CAMs (*Icam-1*, *Icam-2*, and *Vcam-1*) were significantly higher than those in -WT mice (Figure 2A), which is a prerequisite for T-cell infiltration. The endothelium of the brain vessels forms an integral and tightly sealed monolayer, which mainly depends on a series of tight junctions (TJs: occludin, claudin-5, and ZO-1) and adherents junction (AJ: VE-cadherin) expressed on the lateral and contact surfaces of each ECs. Further, they maintain the integrity of the paracellular pathways (Bazzoni and Dejana, 2004; Wallez and Huber, 2008; Tietz and Engelhardt, 2015). In our study, the expression of cell junctions decreased in the brain microvessels of 3.5-month-old -HO mice (Figure 2B); however, the distribution patterns of them, such as localization, morphology, and polarization, remained unchanged in 1-month-old -HO mice (Figure 2C). Exogenous tracers (Evans Blue, 3 kDa, and 70 kDa TMR-dextran) were utilized to directly evaluate BBB permeability in the pre-calcification stage of -HO mice. Under the consistent parameters of fluorescence intensity and exposure time, a slight but significantly increased permeability was observed in the cortex, basal ganglia, and midbrain of the 1.5-month-old pre-calcification -HO mice, compared to -WT (Figure 2D; Supplementary Figure S2). These findings indicate that the increased paracellular permeability made the entry of T-cells into the brain parenchyma more feasible, which was also the cause of neuroinflammation.

### Transcytosis of brain endothelium increased in *Slc20a2*-HO mice

**Figure 3:**
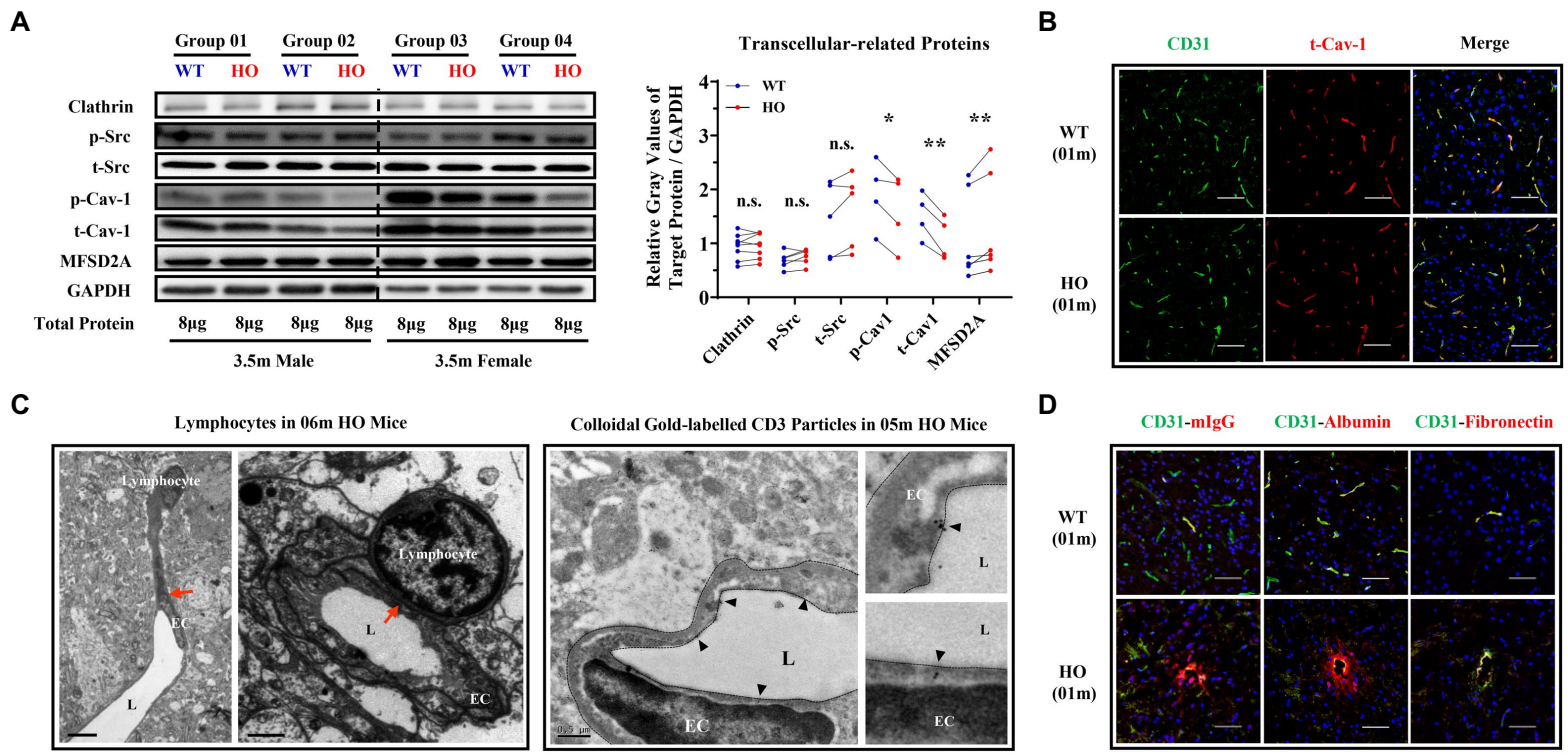
Transcellular Pathway Alterations in The brain endothelium of *Slc20a2*-HO mice. **(A)** Expression of endocytosis- and transcytosis-related proteins in brain microvessels of 3.5-month-old *Slc20a2*-WT and -HO mice. ^**^p < 0.01; ^*^p < 0.05; n.s., not statistically significant. **(B)** Distribution pattern (localization, morphology, and polarization) of total caveolin-1 in 1-month-old mice. **(C)** The fusion of endothelial and lymphocyte cell membranes (red arrows) in 6-month-old -HO mice **(C**, Left**)**. CD3^+^ colloidal gold particles in the endothelial cell membrane of the midbrain in 5-month-old -HO mice. Black triangles indicate gold particles **(C**, Right**)**. EC, endothelial cell; L, vascular lumen. Scale bars, 6 and 1.5 μm **(C**, left**)**; 0.5 μm **(C**, right**)**. **(D)** Endogenous IgG and albumin, rather than fibronectin, leaked into the brains of 1-month-old -HO mice. Scale bar, 40 μm **(B,D)**.

The expression of clathrin and phosphorylated and total Src was not altered, but that of phosphorylated and total Cav-1 was decreased in 3.5-month-old *Slc20a2*-HO mice. Mfsd2a, a key suppressor of caveolae formation and Cav-1 expression, showed slightly increased, coinciding with the decrease of Cav-1 (Figure 3A). The distribution of total Cav-1 remained unchanged in 1-month-old -HO mice (Figure 3B). Transmission electron microscopy (TEM) and immunoelectron microscopy were implemented to investigate whether T-cells traveled directly across BBB-ECs. The fusion of lymphocytes and EC membranes was observed by TEM in the brains of 6-month-old -HO mice; furthermore, immunoelectron microscopy with CD3 antibodies to label T-cells demonstrated that colloidal gold particles were present within brain ECs (Figure 3C), suggesting that CD3^+^ T-cells or endogenous plasma proteins entered the brain parenchyma *via* the transmembrane pathway. Endogenous IgG and albumin (small molecules) could be physically transported across the intact BBB; they were substantially increased in the perivascular spaces of the neurovascular unit (NVU) of 1-month-old pre-calcification -HO mice; macromolecules, such as blood-borne fragments of fibronectin, were undetectable in the brain parenchyma of mice of the same age (Figure 3D).

### Astrocyte and pericyte density and coverage of microvessels remained unchanged

Both astrocytes and pericytes play an essential role in the maintenance of BBB integrity (Zlokovic, 2008). Thus, the distribution pattern and coverage of astrocytes and pericytes in blood vessels were explored using AQP4, PDGF-Rβ, and CD31 antibodies to label astrocytes, pericytes, and vessels, respectively. There were no significant differences in the localization, morphology, polarization, and coverage of astrocytes or pericytes in *Slc20a2*-HO mice compared with -WT mice at the age of either 1 or 3.5 months (Supplementary Figure S3).

### FTY720 Alleviated brain calcification probably By multiple mechanisms in *Slc20a2*-HO mice

**Figure 4:**
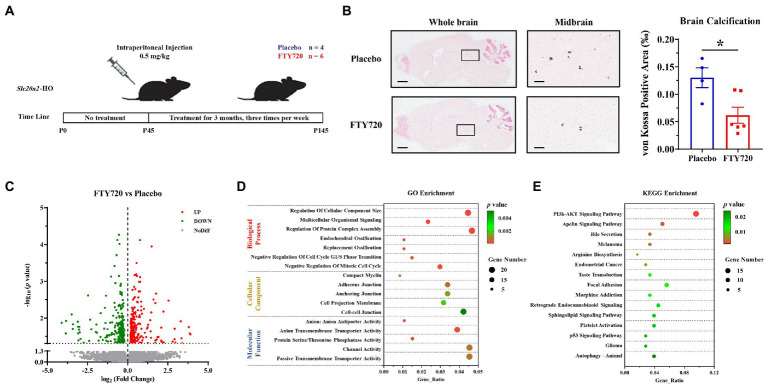
FTY720 alleviated brain calcification and mRNA profile analysis. **(A)** A schematic diagram of the FTY720 administration process. **(B)** Representative images of brain calcification between placebo- and FTY720-treated mice. Area of decreased brain calcification in FTY720-treated mice compared to the placebo group. Scale bars, 1,000 μm, 150 μm. ^*^p < 0.05. **(C)** A volcano plot for the DEGs revealed by RNA-seq, in which 259 and 223 genes were upregulated and downregulated, respectively, with *p* < 0.05 as the cut-off criteria. **(D,E)** GO and KEGG enrichment analyses of the DEGs, with *p* < 0.01 and 0.05 as the cut-off criteria, respectively.

To explore the relationship between T-cell infiltration and calcification, FTY720 was utilized to inhibit the circulation of peripheral T-cells to reduce their CNS infiltration. The procedure for FTY720 administration is shown in Figure 4A. Three months after intraperitoneal administration, brain calcification was significantly reduced in FTY720-treated mice compared with that in the placebo group (FTY720: placebo = 6:4; *p* = 0.0181; Figure 4B). RNA sequencing helped determine the key molecular and biological mechanisms involved in FTY720’s effects on the brain samples in the two groups. A total of 482 DEGs were identified using a filtering criterion of *p* < 0.05, among which 259 and 223 genes were upregulated and downregulated, respectively (Figure 4C; Supplementary Table S3, and GSE218335). GO and KEGG enrichment analyses were performed to ascertain the functional changes in FTY720 treatment, and all terms were divided into the biological process (BP), cellular component (CC), and molecular function (MF) ontologies, as well as KEGG pathways. The DEGs highly enriched in GO_BP were mainly related to the “Ossification” (5 genes) and “Negative Regulation of Cell Cycle” (14 genes) terms; in the GO_CC subset, DEGs were enriched in the “Myelin” (4 genes) and various “Cell Junctions” (24 genes) terms; further, from the GO_MF perspective, the items related to transporter and channel activity (33 genes) constituted most of the enriched terms (Figure 4D; Supplementary Table S3). The “PI3K-AKT Signaling Pathway” was the top one enriched pathway in the KEGG analysis, with eight downregulated and nine upregulated genes. It is worth noting that seven genes in the autophagy pathway were also enriched in KEGG analysis (Figure 4E; Supplementary Table S3).

## Discussion

*Slc20a2*-deficient mice exhibit the clinical symptoms of premature aging that progressively become more severe with age (Beck-Cormier et al., 2019; Ren et al., 2021). Human aging results in physiological brain calcification (Yamada et al., 2013) and a precipitous decline in the adult neural stem or progenitor cells with concomitant cognitive impairment (van Praag et al., 2005; Villeda et al., 2011), similar to the phenotypes of *Slc20a2*-HO mice (Ren et al., 2021). In aging brains, CNS-resident immune cells, such as microglia, increase, and peripheral blood-derived T-cells get recruited (Gemechu and Bentivoglio, 2012). CNS-infiltrating T-cells have been reported to play important roles in neurological diseases and are associated with neuroinflammation. CD4^+^ T-cells can be classified into several functional subgroups based on their surface markers. The T helper (Th)1 and Th17 subgroups secrete high levels of interferon-gamma (IFN-γ) and interleukin-17-related cytokines, which contribute to chronic inflammation. In contrast, Th2 and T regulatory cells are more inclined to modulate the inflammatory responses of brain-resident immune cells (Gonzalez and Pacheco, 2014; Sonar and Lal, 2017; Solleiro-Villavicencio and Rivas-Arancibia, 2018). CNS-infiltrating DNTs were markedly increased in patients and mice with ischemic stroke and positively associated with brain injury (Meng et al., 2019; Kim et al., 2021). In *Pdgfb*^ret/ret^ PFBC mice, the expression of CAMs was upregulated in the cerebral vessels, which was accompanied by more lymphocytes entering the brain parenchyma. Infiltration of peripheral leukocytes into the CNS induces susceptibility to autoimmune inflammation (Torok et al., 2021). Zarb demonstrated that in *Pdgfb*^ret/ret^ mice, a novel microglial subset, calcification-associated microglia, accumulated around vascular calcified regions or in the perivascular space and effectively inhibited brain calcification (Zarb et al., 2021). Brain calcification induces the formation of neurotoxic astrocytes, although the exact functions of these cells remain unknown (Zarb et al., 2019).

The aging brain has a marked susceptibility to circulatory proteins (Conboy et al., 2005; Yang et al., 2020). Endogenous plasma proteins can pass through the BBB *via* a ligand-specific receptor-mediated pathway during adolescence, which changes into non-specific caveolin-mediated transcytosis in the aging brain (Yang et al., 2020). Similarly, increased endocytosis or transcytosis is regarded as an early event of BBB injury in migraine, brain injury, and ischemic stroke (Knowland et al., 2014; Sadeghian et al., 2018). In the brain parenchyma of *Slc20a2*-HO mice, endogenous IgG and albumin gets transported across an impaired BBB and is presented in the perivascular spaces of the NVU (Jensen et al., 2018), suggesting the enhancement of endocytosis or transcytosis. Cav-1 expression is necessary for the formation and transcytosis of caveolae as a structural protein; otherwise, it acts as a potent inhibitor of endothelial nitric oxide synthase activity to negatively regulate paracellular permeability (Schubert et al., 2002). In an ischemic stroke mouse model, Cav-1 deficiency correlated with the increase in the degradation of TJs and the hydrolytic activity of matrix metalloproteinases, thereby enhancing BBB permeability (Choi et al., 2016).

*Slc20a2* mainly expresses in the core components of the NVU, such as SMCs, ECs, pericytes, and the end-processes of astrocytes (Inden et al., 2016; Wallingford et al., 2017; Jensen et al., 2018; Vanlandewijck et al., 2018). Intracellular microcalcifications or tiny calcified granules were detected in the pericytes and astrocytes of *SLC20A2*-related patients (Miklossy et al., 2005) and mice (Jensen et al., 2018); however, neuronal and EC cell death only appeared in severely calcified areas (Kobayashi et al., 1987; Miklossy et al., 2005; Wszolek et al., 2006; Kimura et al., 2016). These findings suggest that, in the *Slc20a2*-HO mice, pericytes and astrocytes have functional defects due to intracellular calcification at an early stage, which then activates endocytosis or transcytosis of BBB-ECs (Alvarez et al., 2013; Keller et al., 2013; Villasenor et al., 2016; Jensen et al., 2018), accompanied by increased BBB permeability from the paracellular pathway.

*Slc20a2* deficiency disturbed brain Pi homeostasis, forming a high-Pi microenvironment (Wallingford et al., 2017) and enhancing the sensitivity of arteriolar SMCs to induce calcification. Pi toxicity is also associated with cellular stress and neuroinflammation (Brown, 2020). *Pdgfb*^ret/ret^ and *Slc20a2*-HO mice exhibited persistent retinitis (Lindblom et al., 2003; Park et al., 2017) and ocular degeneration (Ren et al., 2021), suggesting high-Pi-induced neuroinflammation. XPR1 is the only known Pi exporter in humans, its LOF mutations cause brain calcification, suggesting the essential roles of intracellular Pi homeostasis for maintaining brain health (Legati et al., 2015). *CMPK2* biallelic LOF mutations were linked to neuron mitochondrial defects and the elevated intracellular Pi levels; *Cmpk2*-KO, as well as KI mice bearing patient-derived variants developed brain calcification (Zhao et al., 2022). LOF mutations in cell junctions impair brain ECs, disrupt their integrity, enhance BBB permeability (Saitou et al., 2000; Nitta et al., 2003; Argaw et al., 2009), and cause brain calcification only in humans (Mochida et al., 2010; O'Driscoll et al., 2010; Cen et al., 2020; Schottlaender et al., 2020). *Jam2*-HO mice developed widespread prominent vacuolation instead of brain calcification in the midbrain, cerebral, and cerebellar cortexes (Schottlaender et al., 2020), suggesting that LOF mutations in cell junctions alone are insufficient to cause brain calcification in mice.

Furthermore, there were no apparent impairments in pericyte coverage and BBB integrity in humans (Paucar et al., 2017) and mice (Wallingford et al., 2017; Jensen et al., 2018; Nahar et al., 2020) with *SLC20A2*-related PFBC, which is partially consistent with our results. PDGF-B and its receptor PDGF-Rβ, two more culprits for PFBC (Keller et al., 2013; Nicolas et al., 2013), are mainly and separately expressed in brain ECs and pericytes, and they play an essential role in regulating the functions of BBB-ECs. Only *Pdgfb*^ret/ret^ mice, in which the tissue distribution of PDGF-B is altered, probably due to the loss of a proteoglycan-binding motif, develop brain calcification (Keller et al., 2013; Vanlandewijck et al., 2015; Nikolakopoulou et al., 2017; Nahar et al., 2020). Inexplicably, the EC permeability was higher in the noncalcification-prone than in the calcification-prone brain areas; this region-specific heterogeneity in permeability was not associated with cell junction proteins or pericyte loss (Moura et al., 2017; Villasenor et al., 2017). *MYORG* encodes an endoplasmic reticulum-localized α-glucosidase that is specifically expressed in astrocytes, and its functional defects also cause brain calcification (Vanlandewijck et al., 2018; Yao et al., 2018; Meek et al., 2022). This evidence suggested that pericytes and astrocytes, or the differences in protein glycosylation in their supporting BBB-ECs, might affect the pathogenesis of brain calcification by regulating endocytosis or transcytosis functions (Keller et al., 2013; Villasenor et al., 2017).

FTY720 significantly alleviated autoinflammatory lesions in a *Pdgfb*^ret/ret^ experimental autoimmune encephalomyelitis model and improved the survival rate of the mice (Terry et al., 2016; Torok et al., 2021). In our experiment, FTY720 partially inhibited brain calcification in *Slc20a2*-HO mice, suggesting that CNS-infiltrating T-cells could promote the pathogenesis of brain calcification. Inhibition of the PI3K-AKT pathway can activate autophagy, relieving vascular calcification phenotype *in vitro* and *in vivo*. In CD73-deficient fibroblasts, the increased activation of AKT blocked autophagy and resulted in arterial calcification (Moorhead 3rd et al., 2020). Dexamethasone accelerated the extracellular matrix calcification through activation of AKT signaling and the inhibition of autophagy in osteoarthritis mouse model (Chen et al., 2021). In the db/db mice, 9-PAHSA (a novel endogenous fatty acid) treatment down-regulated Akt–mTOR and activates autophagy in diabetic myocardium, ameliorating carotid vascular calcification (Wang et al., 2021). FTY720 was also reported to play a critical regulatory role in the bone remodeling, particularly in the regulation of osteoclasts, which were closely related to vascular calcification (Ishii et al., 2009; Xiao et al., 2018).

We demonstrated that T-cells infiltrated the CNS of *Slc20a2*-HO mice and that this infiltration rate increased with age. T-cells are likely to enter the brain parenchyma through paracellular leakage. The high restriction of BBB-ECs is a significant barrier to medications intended to cross the BBB. Targeted delivery strategies that induce endocytosis or transcytosis-mediated transport have been extensively studied. Therefore, understanding the immune mechanisms of *Slc20a2*-PFBC can increase our knowledge of disease pathogenesis and guide targeted treatment.

## Data availability statement

The datasets presented in this study can be found in online repositories. The names of the repository/repositories and accession number(s) can be found at: https://www.ncbi.nlm.nih.gov/geo/, GSE218335

## Ethics statement

The animal study was reviewed and approved by the Ethics Committee of The Fourth Affiliated Hospital of Harbin Medical University.

## Author contributions

LS conceived and designed this study. YZ, YR, and YuZ collaboratively conducted all experiments and statistical analyses. YL, CX, ZP, and ZZ provided professional medical guidance, meanwhile, YJ and SQ provided valuable suggestion for interpreting pathophysiological and molecular results. YZ, YR, and LS prepared the original manuscript. YR, YZ, YuZ, and LS checked and revised the grammar, syntax, and logical errors. All authors contributed to the article and approved the submitted version.

## Funding

This study was funded by the National Key R&D Program of China (2020YFA0804000) and the Tou-Yan Innovation Team Program of Heilongjiang Province (2019-15).

## Conflict of interest

The authors declare that the research was conducted in the absence of any commercial or financial relationships that could be construed as potential conflicts of interest.

## Publisher’s note

All claims expressed in this article are solely those of the authors and do not necessarily represent those of their affiliated organizations, or those of the publisher, the editors and the reviewers. Any product that may be evaluated in this article, or claim that may be made by its manufacturer, is not guaranteed or endorsed by the publisher.
