# Supplementary material for: T-cell infiltration in the central nervous system and their association with brain calcification in Slc20a2-deficient mice
Source: Front Mol Neurosci. 2023 Jan 20;16:1073723. doi: 10.3389/fnmol.2023.1073723 (PMC9894888; doi:10.3389/fnmol.2023.1073723)
Supplement: Supplementary file 1 [file Table_1.DOCX]

# Supplementary Table 1 | Antibodies used in this study

| **Antibody (Clone, Species / Isotype)** | **Supplier** | **Cat. Num** | **Dilution** | **Application*** |
| --- | --- | --- | --- | --- |
| CD45 (30-F11), Rat / IgG2b | eBioscience | 14-0451-82 | 1:400 | IF (Frozen) |
| CD3, Rabbit / IgG | Abcam | ab5690 | 1:400/1:50 | IF (Frozen)/IM |
| CD4 (GK1.5), Rat / IgG2b | eBioscience | 14-0041-82 | 1:200 | IF (Frozen) |
| CD8a (53-6.7), Rat / IgG2a | eBioscience | 14-0081-82 | 1:200 | IF (Frozen) |
| CD31, Rabbit / IgG | Abcam | ab28364 | 1:100 | IF (Frozen) |
| CD31 (MEC 13.3), Rat / IgG2a | BD Biosciences | 550274 | 1:100 | IF (Frozen) |
| Immunoglobulin G, Mouse / IgG | Santa Cruz | sc-2025 | 1:100 | IF (Frozen) |
| Albumin, Rabbit / IgG | Proteintech | 16475-1-AP | 1:100 | IF (Frozen) |
| Fibronectin, Rabbit / IgG | Abcam | ab2413 | 1:100 | IF (Frozen) |
| AQP4, Rabbit / IgG | Abomone | AQP-004 | 1:100 | IF (Frozen) |
| CD140b (PDGFRB), Rat / IgG2a | eBioscience | 14-1402-82 | 1:50 | IF (Frozen) |
| CD16/32 (2.4G2), Rat / IgG2b | BD Biosciences | 553142 | 1:67 | FC Blocking |
| FITC-CD45 (30-F11), Rat / IgG2b | eBioscience | 11-0451-82 | 1:100 | FC |
| PerCP-Cy5.5-CD3e (145-2C11), Armenian hamster / IgG | eBioscience | 45-0031-82 | 1:100 | FC |
| PE-CD4 (GK1.5), Rat / IgG2b | eBioscience | 12-0041-82 | 1:300 | FC |
| APC-CD8a (53-6.7), Rat / IgG2a | eBioscience | 17-0081-82 | 1:100 | FC |
| FITC-Rat IgG2b (eB149/10H5) | eBioscience | 11-4031-82 | 1:100 | Isotype Control |
| PerCP-Cy5.5-Armenian hamster IgG (eBio299Arm) | eBioscience | 45-4888-80 | 1:100 | Isotype Control |
| PE-Rat IgG2b (eB149/10H5) | eBioscience | 12-4031-82 | 1:300 | Isotype Control |
| APC-Rat IgG2a (eBR2a) | eBioscience | 17-4321-81 | 1:100 | Isotype Control |
| VE-Cadherin, Goat / IgG | R&D Systems | AF1002 | 1:5000/1:100 | WB/IF (Frozen) |
| Occludin (OC-3F10), Mouse / IgG1 | Invitrogen | 33-1500 | 1:1000/1:100 | WB/IF (Frozen) |
| Claudin-5 (4C3C2), Mouse / IgG1 | Invitrogen | 35-2500 | 1:1000/1:100 | WB/IF (Frozen) |
| ZO-1 (ZO1-1A12), Mouse / IgG1 | Invitrogen | 33-9100 | 1:1000/1:100 | WB/IF (Frozen) |
| Phospho-Src Family (Tyr416), Rabbit / IgG | CST | 2101S | 1:1000 | WB |
| Src (36D10), Rabbit / IgG | CST | 2109S | 1:1000 | WB |
| t-Clathrin, Mouse / IgG1 | BD Bioscience | 610499 | 1:1000 | WB |
| Caveolin-1 (Py14), Mouse / IgG1 | BD Bioscience | 611338 | 1:2000 | WB |
| Caveolin-1 (D46G3), Rabbit / IgG | CST | 3267T | 1:1000 | WB/IF (Frozen) |
| Mfsd2a, Rabbit / IgG | Invitrogen | PA5-21049 | 1:1000 | WB |
| GAPDH (14C10), Rabbit / IgG | CST | 2118S | 1:4000 | WB |

***** IF = Immunofluorescence, IM = Immunoelectron Microscopy, FC = Flow Cytometry, WB = Western Blotting
