# Supplementary material for: T-cell infiltration in the central nervous system and their association with brain calcification in Slc20a2-deficient mice
Source: Front Mol Neurosci. 2023 Jan 20;16:1073723. doi: 10.3389/fnmol.2023.1073723 (PMC9894888; doi:10.3389/fnmol.2023.1073723)
Supplement: Supplementary file 2 [file Table_2.docx]

**Supplementary Table 2 | Primers for Real-time Quantitative PCR**

| **Mouse Gene** | **Transcript ID** | **Primer Sequence (from 5’ to 3’)** | **Tm** | **Exon Spanning,**  **Amplicon Length** |
| --- | --- | --- | --- | --- |
| *Icam-1* | NM_010493.3 | FOR: GTGGCGGGAAAGTTCCTG | 60.0 | YES, 170 bp |
|  |  | REV: CGTCTGCAGGTCATCTTAGGAG |  |  |
| *Icam-2* | NM_010494.2 | FOR: ATCAACTGCAGCACCAACTG | 60.0 | YES, 215 bp |
|  |  | REV: ACTTGAGCTGGAGGCTGGTA |  |  |
| *Vcam-1* | NM_011693.3 | FOR: AGTTGGGGATTCGGTTGTTC | 60.0 | NO, 107 bp |
|  |  | REV: CATTCCTTACCACCCCATTG |  |  |
| *Sele* / *E-selectin* | NM_011345.2 | FOR: AGCTACCCATGGAACACGAC | 60.0 | YES, 199 bp |
|  |  | REV: ACGCAAGTTCTCCAGCTGTT |  |  |
| *Selp* / *P-selectin* | NM_011347.2 | FOR: GTCCACGGAGAGTTTGGTGT | 60.0 | YES, 241 bp |
|  |  | REV: AAGTGGTGTTCGGACCAAAG |  |  |
| *Hprt* | NM_000194.3 | FOR: CTTCCTCCTCAGACCGCTTT | 60.0 | YES, 146 bp |
|  |  | REV: TTTTCCAAATCCTCGGCATA |  |  |
