# Supplementary figures and images for: T-cell infiltration in the central nervous system and their association with brain calcification in Slc20a2-deficient mice
Source: Front Mol Neurosci. 2023 Jan 20;16:1073723. doi: 10.3389/fnmol.2023.1073723 (PMC9894888; doi:10.3389/fnmol.2023.1073723)

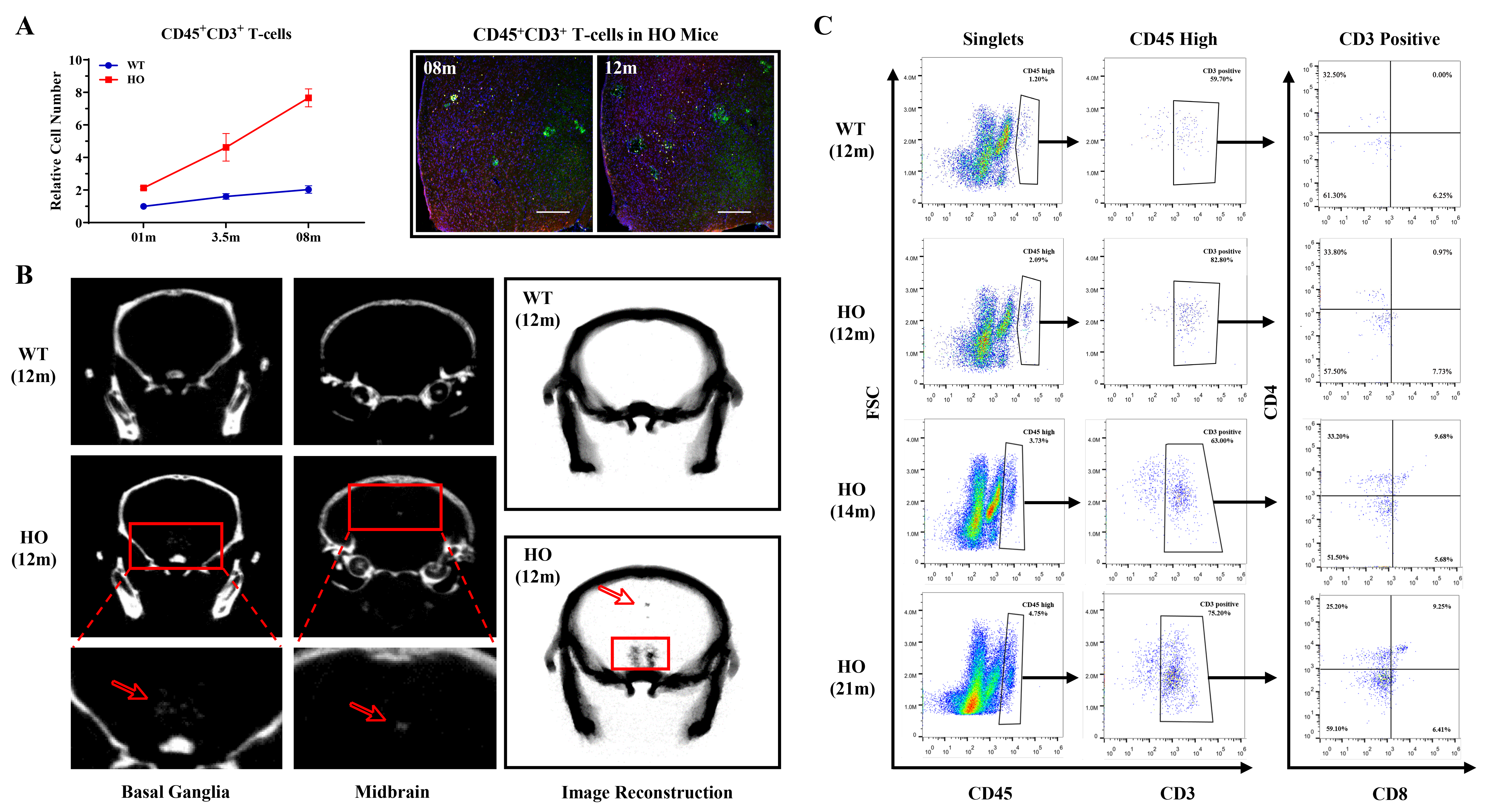

Supplement: Supplementary file 4 [file Image_1.TIF]

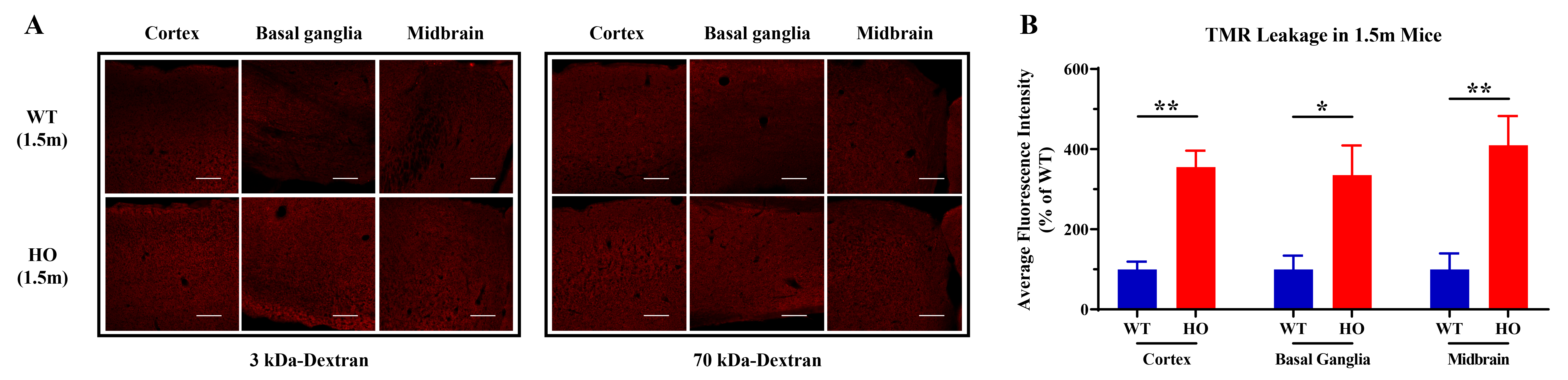

Supplement: Supplementary file 5 [file Image_2.TIF]

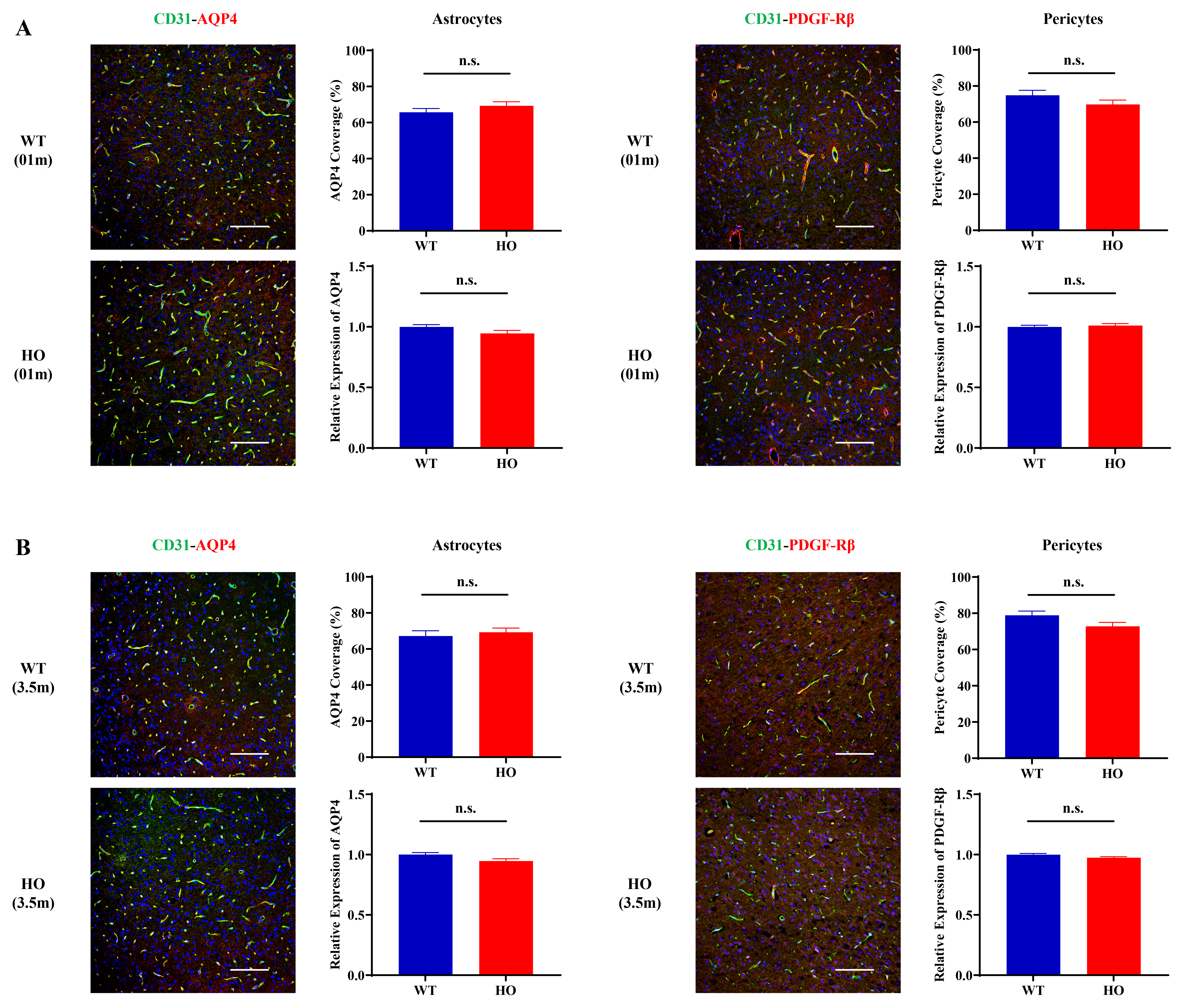

Supplement: Supplementary file 6 [file Image_3.TIF]
